# Neomycin Exhibits Immunomodulatory and Antiviral Activity Against Influenza B Virus

**DOI:** 10.3390/v18040444

**Published:** 2026-04-07

**Authors:** Ekaterina Romanovskaya-Romanko, Marina Plotnikova, Anna-Polina Shurygina, Marina Shuklina, Sergey Klotchenko, Zhanna Buzitskaya, Dmitry Lioznov, Marina Stukova

**Affiliations:** Smorodintsev Research Institute of Influenza, Ministry of Health of the Russian Federation, Saint-Petersburg 197022, Russia

**Keywords:** antiviral, neomycin, immunomodulation, influenza B, mucosal immunity

## Abstract

Viral infections remain a global public health challenge. Stimulating the innate immune system is a potent therapeutic strategy that promotes pathogen clearance, directly impacting disease severity and clinical outcomes. Interferons and interferon-stimulated genes (ISGs) are critical components of this antiviral defense system. Neomycin, an aminoglycoside antibiotic, can induce ISG expression and help establish an antiviral state. In this study, we demonstrated that neomycin induces the production of pro-inflammatory cytokines (IL1β, TNFα, IL6, GM-CSF, and IFN-γ) in peripheral mononuclear blood cells (PBMCs) and activates key antiviral ISGs, including MxA, OAS1, and IRF7. The protein expression profiles elicited by neomycin were comparable to those induced by poly(I:C). Intranasal delivery of neomycin to CBA and BALB/c mice induced various ISGs in both the respiratory tract and splenic tissues. Prophylactic administration of neomycin significantly inhibited influenza B virus replication in the lung and nasal turbinates of CBA mice in a sublethal infection model. Overall, our data suggest that neomycin, when used prophylactically alone or combined with other antiviral strategies, shows considerable potential for the attenuation of influenza B virus infections.

## 1. Introduction

Viral infections remain a serious threat to global public health [1]. Annually, seasonal epidemics caused by influenza A and B viruses alone lead to 3–5 million severe cases and 290,000–650,000 respiratory deaths worldwide [2]. As the primary tool for preventing viral infections, vaccines do not always provide effective protection against newly emerging antigenic variants because many viruses show high variability and spread rapidly. In this regard, antiviral drugs become a promising approach to effectively controlling viral infections [3].

In 2019, the World Health Organization released a Global Influenza Strategy for 2019–2030 that prioritizes the development and implementation of new approaches to influenza treatment and prevention. This includes antiviral drugs based on immunomodulators, which have a wide range of effects against various genotypes or subtypes of the virus, as their targeted inhibitory action does not directly affect viral proteins [4]. The use of immunomodulatory drugs against various diseases is a promising and actively developing therapeutic approach. Market analysts predict steady growth in the immunomodulator sector, with cytokine modulators emerging as one of the fastest-growing segments, driven by advances in biotechnology and personalized medicine [5].

Neomycin, an aminoglycoside antibiotic, has previously been shown to exhibit antiviral activity against DNA and RNA viruses. It is formulated for topical use in creams, ointments, aerosols, and eye drops at active substance concentrations of 0.5–3.3%. Historically, neomycin was also used for partial intestinal decontamination prior to gastrointestinal surgery and as part of combination therapy for gastrointestinal disorders (enteritis, enterocolitis, hepatic encephalopathy, and hypercholesterolemia). Systemic use, however, is associated with significant adverse effects, including nephrotoxicity, ototoxicity, seizures after intracisternal injection, and inhibition of neuromuscular and ganglionic transmission [6]. Consequently, neomycin is currently indicated primarily for topical application. Its antimicrobial mechanism involves binding to the prokaryotic 30S ribosomal subunit, thereby inhibiting bacterial protein synthesis [7].

Neomycin induces interferon-stimulated gene (ISG) upregulation in the nasal cavity and vaginal mucosa in various animal species, facilitating the establishment of an antiviral state in the organism. Importantly, the antiviral effect of neomycin against influenza A virus and SARS-CoV-2 is IFN-independent [8]. However, it remains unclear how neomycin affects immune cells and their role in forming the antiviral state. This study investigated the antiviral properties of the aminoglycoside antibiotic neomycin against influenza B virus infection in two inbred mouse strains and sought to elucidate the underlying molecular mechanisms of action. We administered neomycin intranasally as either a prophylactic or therapeutic agent to evaluate whether it can protect CBA and BALB/c mice against lethal influenza B virus (B/Malaysia/2506/04) infection or attenuate disease severity in a sublethal infection model. We also assessed the immunomodulatory potential of neomycin in human PBMCs and in vivo.

## 2. Materials and Methods

### 2.1. Cell Culture and Challenge Virus

Madin–Darby canine kidney cells (MDCKs, #FR-58, IRR) were cultivated in DMEM supplemented with 10% fetal bovine serum (Biolot, Saint-Petersburg, Russia). PBMCs were isolated from buffy coats obtained from healthy anonymous volunteers at the blood transfusion station, Pavlov First Saint Petersburg State Medical University (Saint-Petersburg, Russia). All donors provided written informed consent, and the study was approved by the Local Ethics Committee of the Smorodintsev Research Institute of Influenza (Approval #192, 16 October 2023). PBMCs were isolated using the standard Ficoll (Biolot, Russia) density gradient separation technique and then frozen for storage. Human PBMCs were cultivated in RPMI-1640 (Biolot, Russia) medium supplemented with 10% FBS HI (Thermo Scientific, Waltham, MA, USA) and 1% anti-anti (Thermo Scientific, USA).

The mouse-adapted influenza strain B/Malaysia/2506/04 of the B/Victoria lineage from the collection of the Smorodintsev Research Institute of Influenza was used as the challenge virus. The virus was propagated in 10-day chicken embryos at 32 °C for 3 days.

### 2.2. Animals

Several 6–8 weeks old female BALB/c (n = 75) and CBA (n = 85) mice were purchased from the Shemyakin–Ovchinnikov Institute of Bioorganic Chemistry of the Russian Academy of Sciences (Nursery for the Laboratory Animals, Puschino, Russia). A total of 45 BALB/c and 45 CBA mice were used for the ISG induction and lethal challenge experiments; a total of 40 CBA mice were used for the low-dose challenge and pulmonary cellular response evaluation. An additional 20 CBA and 30 BALB/c mice were used for challenge virus dose selection. The animals were kept in standard conditions in accordance with the provisions of Directive 2010/63/EU, the federal guidelines, and the institutional policies of the Smorodintsev Research Institute of Influenza. All experiments involving laboratory animals were approved by the Bioethics Committee of the Smorodintsev Research Institute of Influenza (protocols No. 17, dated 10 July 2025, and No. 02, dated 2 February 2026).

### 2.3. Virus Infection

Under light ether anesthesia, the animals were intranasally inoculated with 30 µL of the challenge virus at a dose of 2 MLD_50_ (50% mouse lethal dose, lethal challenge) or, in the case of the sublethal challenge, with 15 µL at a dose of 2.7 log_10_ EID_50_ using a pipette tip. Sterile Dulbecco’s phosphate-buffered saline (DPBS; Biolot, Russia) was used to prepare the challenge virus. The MLD_50_ was determined using virus stock titration in BALB/c mice (Supplementary Figure S1).

### 2.4. Antibiotic Treatment

Neomycin sulfate (Agropharm, Voronezh, Russia) was dissolved in sterile water at a concentration of 80 mg/mL and used immediately without storage. PBS was used as the vehicle control. A total of 25 μL of the antibiotic or vehicle solution was administered dropwise into the nasal cavity using a pipette tip to animals under light ether anesthesia.

### 2.5. Measurement of Viral Infectious Activity

Mice were sacrificed by cervical dislocation at specified time points. Whole lungs were isolated, placed in tubes with 1 mL of PBS, and homogenized using TissueLyser II (Qiagen, Germantown, MD, USA). Infectious influenza virus titers were determined by limiting the dilution assay on one-day-old monolayers of MDCK cells. Ten-fold serial dilutions of the virus were prepared in DMEM (Biolot, Russia) supplemented with 1% antibiotic–antimycotic (Thermo Scientific, USA) and 1 μg/mL TRCK–trypsin (Sigma-Aldrich, St. Louis, MO, USA). Each dilution was analyzed in quadruplicate. The 50% tissue culture infectious dose (TCID50) was calculated according to the Reed and Muench method and expressed in log_10_ [9].

### 2.6. RNA Isolation and RT-qPCR

Total RNA from mouse organs was isolated using the ExtractRNA reagent (Evrogen, Moscow, Russia) according to the manufacturer’s instructions. Immediately after euthanasia, lung and spleen tissue samples were placed in tubes containing the IntactRNA fixation solution (Evrogen, Russia), incubated for 1 h at room temperature, and stored at −20 °C for no more than a week until RNA extraction.

RNA from PBMCs was extracted using the RNA Solo kit (Evrogen, Russia), which includes a DNase treatment step. The cell monolayer was pre-washed with DPBS, and 200 μL of lysis buffer was added to the plate wells. RNA isolation was performed according to the manufacturer’s instructions, and then RNA concentration and integrity were analyzed using a NanoDrop ND-1000 spectrophotometer (Thermo Scientific, USA). RNA samples were stored at −80 °C.

#### 2.6.1. Reverse Transcription

RNA isolated from mouse tissues was pre-treated with RQ1 RNase-Free DNase (Promega, Madison, WI, USA). The reverse transcription reaction was performed using 1 μg of RNA, 0.5 μg of oligo-dT16 primers (DNA synthesis, Moscow, Russia), and the RNAscribe RT kit (BioLabMix, Novosibirsk, Russia). Complementary DNA synthesis was carried out at 50 °C for 50 min. The enzyme was inactivated at 80 °C for 5 min. cDNA products were diluted (1:2) and stored at −20 °C until further use in PCR.

#### 2.6.2. PCR Analysis

Real-time PCR assays were performed using the GenTier 96E PCR Cycler (TianLong, Xi’an, China). Multiplex qPCR was run in 25 μL final reaction volumes containing 12.5 μL of the 2x BioMaster HS-qPCR mix (BioLabMix, Russia) and 5 μL of cDNA. The reaction mixture contained 300 nM of each primer and 200 nM of the TaqMan probe (final concentrations). The PCR conditions were 95 °C for 5 min, followed by 40 amplification cycles (95 °C for 10 s, 58 °C for 10 s, and 72 °C for 10 s).

The primer and probe sequences used to assess gene expression are listed in Supplementary Table S1 (mouse tissues) and Table S2 (human cells).

The Ct values were normalized against several housekeeping genes in mice (average of *Hprt1*, *GAPDH*, *Ubc*, and *Rplp0*) or *GAPDH* in human cells and then compared against biological controls (untreated mice or cells) using the ΔΔCt method. Fold expression was calculated assuming a doubling of efficiency (2) per cycle (fold expression = 2^−ΔΔCt^).

### 2.7. Gene Expression and Cytokine Production in Neomycin-Treated PBMCs

Human PBMCs obtained from two healthy donors were incubated with 2.0 mg/mL neomycin sulfate or polyinosinic:polycytidylic acid (poly(I:C)) at a concentration of 10 µg/mL at 37 °C for 24 h in standard medium. To minimize the risk of nonspecific stimulation, cell samples from different donors were not pooled. For each donor, 4 biological replicates (2 × 10^6^ cells for each sample) were prepared, resulting in n = 8 per experimental group. The cytokine concentration in supernatants was determined using a multiplex flow cytometry panel, and gene expression was measured in lysed cells by RT-qPCR.

### 2.8. Cytokine Concentrations

The commercial LEGENDplex™ Human Anti-Virus Response Panel (Biolegend, San Diego, CA, USA) was used to simultaneously measure the concentrations of IFN-λ1 (IL29), IL1β, IL6, TNF-α, IP10, IL8, IL12p70, IFN-α2, IFN-λ2 (IL28A), GM-CSF, IFN-β, IL10, and IFN-γ. According to the manufacturer’s instructions, 25 µL of the buffer solution and 25 µL of antibody-labeled particles were added to 25 µL of the sample or a serial dilution of the standard in a 96-well V-bottom plate. The samples were then incubated on a shaker (800 rpm) for 2 h at room temperature. Following centrifugation (300 rpm for 7 min at room temperature) and washing, 25 µL of biotinylated secondary antibodies was added to the wells and incubated for another 1 h under the same conditions. Next, 25 µL of the streptavidin–phycoerythrin conjugate was added to the wells and incubated for another 30 min. Before analysis, the samples were centrifuged once, the supernatants were decanted, and the pellets were resuspended in 100 µL of DPBS. Data collection was performed on a CytoFLEX flow cytometer (Beckman Coulter, Brea, CA, USA), and the results were analyzed using Kaluza 2.2 software (Beckman Coulter, USA).

### 2.9. Evaluation of Viral Load by RT-PCR

Viral RNA from mouse organ homogenates was isolated using the RNeasy Mini Kit (Qiagen, Venlo, The Netherlands). RT-qPCR was performed using the BioMaster RT-PCR-Extra (2×) reagent kit (Biolabmix, Russia), InfB-F (TCCTCAAYTCACTCTTCGAGCG) and InfB-R (CGGTGCTCTTGACCAAATTGG) primers, and the InfB-P oligonucleotide probe ((FAM)-CCAATTCGAGCAGCTGAAACTGCGGTG-(BHQ1)). The RT-PCR conditions were as follows: 50 °C for 60 min, 95 °C for 5 min, followed by 40 amplification cycles of 95 °C for 5 s, 57 °C for 10 s, and 68 °C for 5 s. Amplification and fluorescence detection were performed using a GenTier 96E PCR Cycler (TianLong, China).

### 2.10. Flow Cytometry

Lungs were harvested from mice 24 and 48 h after intranasal administration of neomycin or PBS to assess the dynamics of innate immune cell populations. Harvested tissues were mechanically dissociated and digested using collagenase/DNase (Sigma, St Louis, MO, USA). The resulting single-cell suspensions were filtered through 70 µm cell strainers, followed by erythrocyte lysis using RBC Lysis Buffer (BioLegend, San Diego, CA, USA). For immunophenotyping, aliquots of 1 × 10^6^ cells in 100 µL were stained with a panel of fluorochrome-conjugated antibodies: CD11b-PE/Cy7, CD11c-PE, MHCII-Alexa488, CD103-PerCP-Cy5.5, CD45-APC/Cy7, CD64-BV421, and CD24-BV510 (all from BioLegend, USA). This panel enabled identification of alveolar macrophages, interstitial macrophages, monocytes, neutrophils, and two subsets of dendritic cells (DC1 and DC2). To minimize non-specific antibody binding, the TrueStain reagent (BioLegend, USA) was added prior to staining. Data were acquired on a CytoFLEX flow cytometer (Beckman Coulter, USA) and analyzed using Kaluza 2.2 software (Beckman Coulter, USA). Cell populations were identified using the gating strategy described by Yu et al. [10], as illustrated in Supplementary Figure S1.

### 2.11. Statistical Analyses

GraphPad Prism 9.0 (GraphPad Software, Inc., La Jolla, CA, USA) was used for statistical analyses. Data are presented as the mean ± standard deviation (SD) or standard error of the mean (SEM), as indicated. Groups were compared using one-way ANOVA followed by Tukey’s multiple comparison test, the Kruskal–Wallis test, or the Mann–Whitney U test, as specified in figure captions. Differences in survival times were assessed using the log-rank test. A *p*-value < 0.05 was considered statistically significant.

## 3. Results

### 3.1. Neomycin Induces Cytokines and Pro-Inflammatory Factors in Human PBMCs Similar to Poly(I:C)

First, we assessed how neomycin affects pro-inflammatory factors and cytokines in human peripheral blood immune cells. To evaluate gene expression changes in response to neomycin, PBMCs from two healthy donors were stimulated with neomycin sulfate at a concentration of 2.0 mg/mL for 24 h. Mock-treated control cells (CCs) and cells treated with poly(I:C) at a concentration of 10 µg/mL were used as controls.

**Figure 1:**
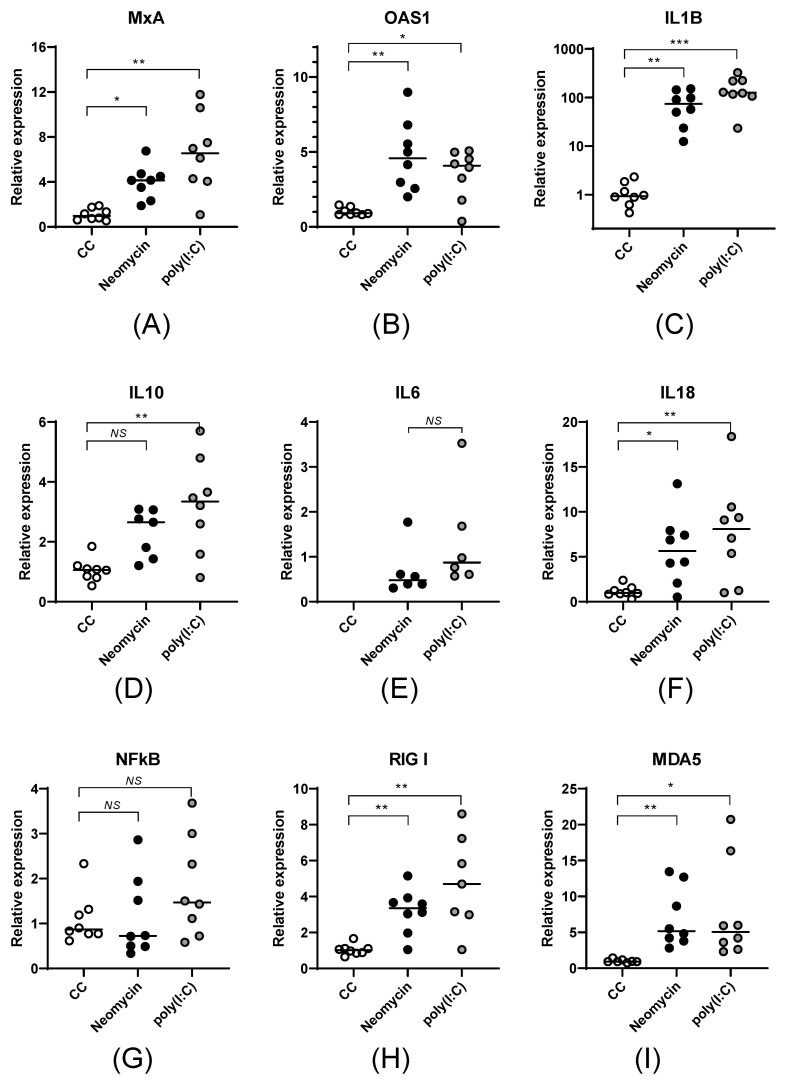
Neomycin stimulation of PBMCs activates the expression of cytokines, transcription factors, and antiviral proteins. mRNA levels of *MxA* (**A**), *OAS1* (**B**), *IL1β* (**C**), *IL10* (**D**), *IL6* (**E**), *IL18* (**F**), *NFkB* (**G**), *RIGI* (**H**), *MDA5* (**I**) were measured in PBMCs after 24 h of stimulation with neomycin (2.0 mg/mL) or poly(I:C) (10 μg/mL). The relative mRNA expression is presented as the mean value (horizontal lines) and individual measurements (dots). To assess the statistical significance of differences in normalized ΔCt values, the non-parametric Kruskal–Wallis test followed by the Dunn correction for multiple comparisons was used (NS—*p* > 0.05; *—*p* < 0.05; **—*p* < 0.01; ***—*p* < 0.001).

At the transcription level, the quantitative and qualitative expression profiles of the studied genes were similar in neomycin- and poly(I:C)-treated cells. Neomycin stimulation led to a statistically significant increase in the mRNA levels of the antiviral genes *MxA* (Figure 1A, *p* < 0.05) and *OAS1* (Figure 1B, *p* < 0.01) and the cytokines *IL18* (Figure 1F, *p* < 0.05), *IL1β* (more than 50 times, Figure 1C, *p* < 0.01), and *IL10*, as well as the induction of *IL6* mRNA. Neomycin caused a 4-5-fold increase in the mRNA levels of the cytosolic sensors *RIGI* (Figure 1H, *p* < 0.01) and *MDA5* (Figure 1I, *p* < 0.01). Neither neomycin nor poly(I:C) affected the expression of the transcription factor *NFkB*.

Next, we measured the concentrations of IFN-λ1 (IL29), IL1β, IL6, TNF-α, IP10, IL8, IL12p70, IFN-α2, IFN-λ2 (IL28A), GM-CSF, IFN-β, IL10, and IFN-γ in cell media to determine cytokine production by PBMCs in response to neomycin stimulation. Neomycin, as well as poly(I:C), activated type III IFN production, with IFN II also showing a tendency to a slight increase compared to control intact cells (CCs). Neomycin had no impact on type I IFN production (Figure S2), which is consistent with data obtained by other research groups [8,11].

**Figure 2:**
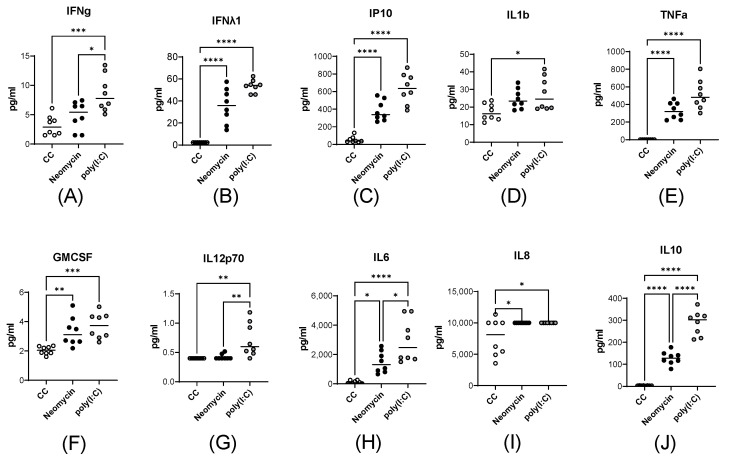
Neomycin activates cytokine production in peripheral blood mononuclear cells (PBMCs). Protein production of IFNγ (**A**), IFN-λ1 (**B**), IP10 (**C**), IL1β (**D**), TNF-α (**E**), GM-CSF (**F**), IL12p70 (**G**), IL6 (**H**), IL8 (**I**), IL10 (**J**) was measured in the supernatant of PBMC cell media after 24 h of neomycin (2 mg/mL) or poly(I:C) (10 μg/mL) stimulation. The data are presented as the mean cytokine concentrations (pg/mL, horizontal lines) and individual values for each biological replicate (dots). Statistical significance was assessed by the nonparametric Kruskal–Wallis test followed by the Dunn correction for multiple comparisons (*—*p* < 0.05; **—*p* < 0.01; ***—*p* < 0.001, ****—*p* < 0.0001).

Neomycin caused a significant increase in the pro-inflammatory cytokines IP10 (Figure 2C, *p* < 0.0001), TNF-α (Figure 2E, *p* < 0.0001), IL6 (Figure 2H, *p* < 0.05), and IL8 (Figure 2I, *p* < 0.05) but had modest effect on GM-CSF (Figure 2F, *p* < 0.01), IL1β (Figure 2D, *p* ≥ 0.05), and IL12p70 (Figure 2G, *p* ≥ 0.05). Importantly, PBMCs increased the production of the anti-inflammatory cytokine IL10 by 100-fold when stimulated with neomycin (Figure 2J, *p* < 0.0001).

Thus, neomycin stimulation of PBMCs led to intensified IL1β, IL6, and IL10 expression at the transcriptional level and IL6, and IL10 production at the translational level. In general, the functional profile of cytokine production induced in response to neomycin shared several features with poly(I:C)-induced cytokine production.

### 3.2. Intranasal Neomycin Treatment Induces an ISG Response in Lung and Spleen Tissues in Mx1-Deficient Mice

Since neomycin demonstrated a significant immunomodulatory effect in human PBMCs in vitro, we evaluated its action in two mouse strains lacking functional Mx1 protein and several other antiviral factors, as deficiencies in key antiviral factors can lead to inadequate immune responses.

**Figure 3:**
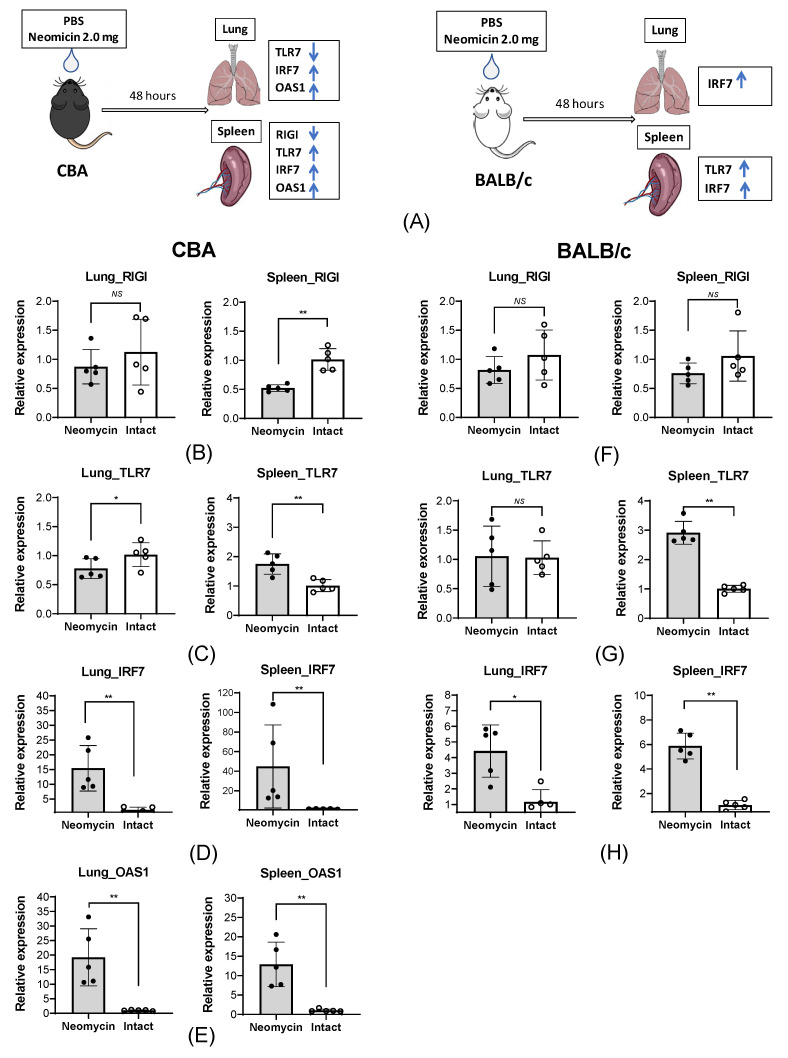
Intranasal administration of neomycin affects the expression of the intracellular RIGI and TLR7 receptors and ISGs in the lungs and the spleen of CBA and BALB/c mice. (**A**) Design of the experiment. BALB/c and CBA mice were intranasally administered with 25 µL of neomycin sulfate at a dose of 2 mg (n = 5) or the equivalent volume of PBS (n = 5) for three days. Then, 48 h after the last administration, tissue samples were collected to evaluate gene expression. The data is presented as the mean relative expression level ± SD, with dots indicating individual values for each animal. (**B**–**E**) The relative expression of RIGI (**B**), TLR7 (**C**), IRF7 (**D**), and OAS1 (**E**) in the lungs and spleen of CBA mice. (**F**–**H**) The relative expression of RIGI (**F**), TLR7 (**G**), and IRF7 (**H**) in the lungs and spleen of BALB/c mice. The Mann–Whitney test was used to assess the statistical significance of differences in normalized ΔCt values (NS—*p* > 0.05; *—*p* < 0.05; **—*p* < 0.01).

Specifically, we assessed the expression of the RIGI, TLR7, IRF7, and OAS1 genes in the lungs and the spleen—the largest lymphoid organ—in response to intranasal administration of neomycin. CBA and BALB/c mice were intranasally inoculated with 2 mg/25 µL of neomycin daily for three consecutive days. PBS was used as a control vehicle. On day 2 after the last neomycin administration, samples of lung and spleen tissues were collected from animals for mRNA isolation and subsequent RT-qPCR (Figure 3A).

Our data show that intranasal administration of neomycin in intact mice of both lines affects the expression of the studied genes not only in the respiratory tract but also at the systemic level.

In CBA mice, a statistically significant upregulation of the pro-inflammatory genes OAS1 (Figure 3E) and IRF7 (Figure 3D) in response to neomycin was observed in both the lungs and the spleen. In contrast, RIGI expression (Figure 3B) in the spleen significantly decreased; a similar trend was observed in the lungs, although the difference did not reach statistical significance. The mRNA level of the endosomal TLR7 receptor (Figure 3C) in CBA animals decreased slightly in the lungs but increased in the spleen.

In BALB/c mice, the mRNA levels of the pro-inflammatory factor IRF7 (Figure 3H) in both organs changed similarly to those in CBA mice. RIGI expression (Figure 3F) in the lungs and spleens showed a decreasing trend in response to neomycin administration, although this trend was not statistically significant. As in CBA mice, the TLR7 mRNA levels (Figure 3G) in the spleen of BALB/c mice increased in response to neomycin. It is important to note that, unlike in CBA mice, no OAS1 mRNA was detected in BALB/c animals.

### 3.3. A Single Prophylactic Intranasal Administration of Neomycin Provides Different Levels of Antiviral Protection Against the Influenza B Virus Lethal Challenge in CBA and BALB/c Mice

To determine whether neomycin has antiviral properties against influenza B virus, we used a mouse model of lethal influenza infection with the Victoria-lineage strain B/Malaysia/2506/04.

We investigated how prophylactic and therapeutic intranasal administration of neomycin impacts the outcome of a lethal influenza B virus infection in Mx1-deficient CBA and BALB/c mice. It is known that most mouse strains do not carry functional copies of Mx1, the key factor mediating immunity to influenza viruses. Additionally, some inbred lines may have defects in ISGs (such as OAS, IRF7) and IFNs that are crucial for the establishment of the host’s antiviral status [12,13].

**Figure 4:**
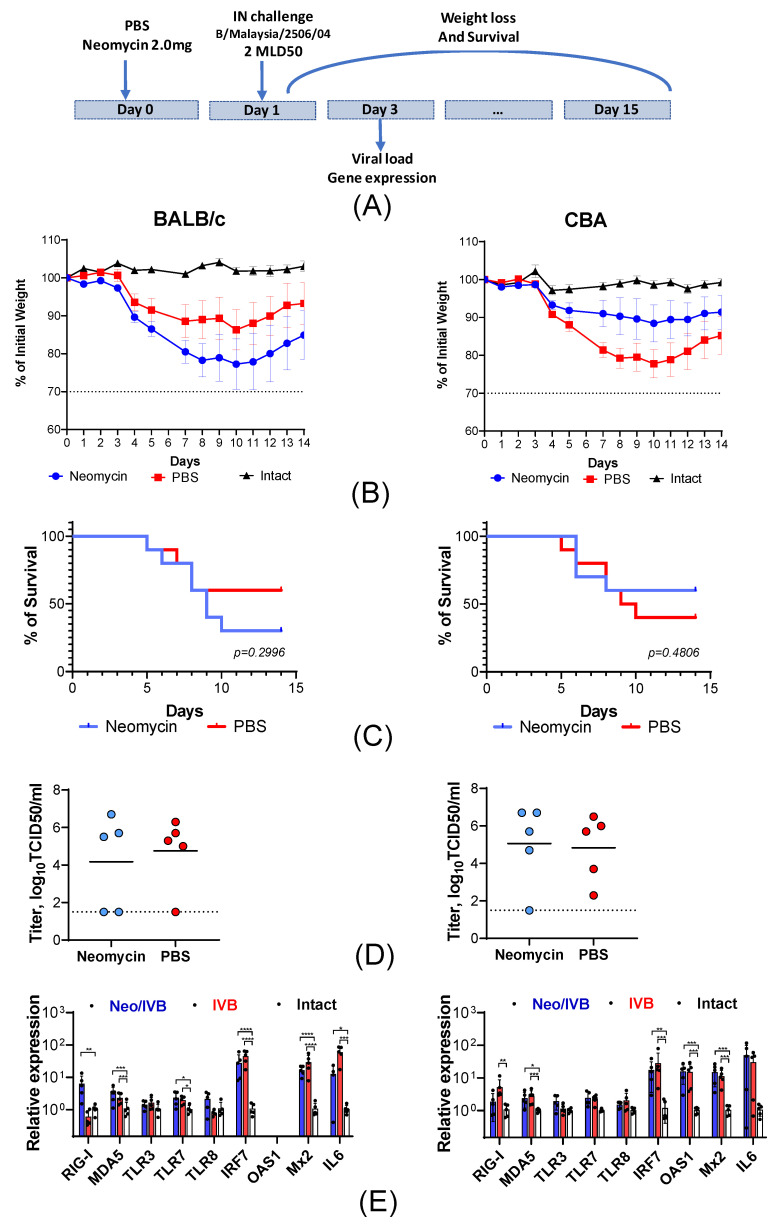
Prophylactic administration of neomycin in CBA and BALB/c mice is associated with varying degrees of influenza B virus infection severity. (**A**) Design of the experiment. CBA and BALB/c mice were given a single intranasal dose of 2 mg of neomycin or PBS in a volume of 25 µL and intranasally infected with influenza virus B/Malaysia/2506/04 (2 MLD_50_) 24 h later. The animals’ body weight (**B**) and survival (**C**) were monitored daily for 14 days after infection. If an animal lost more than 30% of its initial body weight, it was sacrificed in accordance with ethical guidelines. On day 3 of the study, the infectious virus titers (**D**) and the expression of intracellular nucleic acid sensors and pro-inflammatory factors (**E**) were measured in lung tissue. Statistical significance was assessed by ordinary one-way ANOVA followed by the Holm-Sidak’s multiple comparisons test (*—*p* < 0.05; **—*p* < 0.01; ***—*p* < 0.001, ****—*p* < 0.0001).The dotted line indicates the detection limit.

In the prophylactic regimen, 25 µL of the neomycin solution was delivered to the lower respiratory tract 24 h prior to infection. This induced an antiviral effect in situ at the site of infection, where the underlying pathological process develops (Figure 4A). We found that a single prophylactic administration of neomycin resulted in a disease of varying severity in mice from both studied strains. BALB/c mice treated with neomycin (Neo) experienced a more severe course of influenza B virus-induced illness compared to animals that received an equivalent volume of PBS (PBS), which was accompanied by a significantly higher body weight loss. The maximum weight loss on day 10 after infection was 23% of the initial body weight in the Neo group and 14% in the PBS group (Figure 4B). Mortality in BALB/c mice was also higher when neomycin was used compared to PBS (60% vs. 30%; Figure 4C).

The opposite pattern was observed in CBA mice, as the PBS group developed more severe infection compared to the Neo group. The most pronounced body weight loss was also observed in the control animals, reaching 23% of the initial weight, whereas it did not exceed 12% in mice that received neomycin (Figure 4B). The mortality rate was 40% in the Neo group and 60% in the PBS group (Figure 4C).

To evaluate the effect of neomycin on influenza B virus replication during the early stages of infection, we measured the viral load in the lungs of CBA and BALB/c mice on day 2 after infection. Neomycin treatment did not significantly affect the viral load in the lungs, with virus titers ranging from 4.0 to 5.0 TCID_50_/mL (Figure 4D).

Next, to investigate how neomycin administration affects the severity of the inflammatory response, we analyzed the expression of intracellular nucleic acid sensors and pro-inflammatory factors in the lungs on day 2 post-infection. It was shown that neomycin did not affect the expression levels of endosomal sensors (TLR3, TLR7, and TLR8) and MDA5 (Figure 4E) in infected animals of both mouse lines. Infected BALB/c mice treated with neomycin showed a significant 10-fold increase in mRNA levels for the cytosolic sensor RIGI. Infection naturally stimulated several ISGs, including IRF7, Mx2, and IL6. At the same time, neomycin did not produce any cumulative effect on the expression of these genes (Figure 4E). Interestingly, no OAS1 mRNA was detected in either infected or intact BALB/c mice, although it was present in intact CBA mice and increased significantly after infection (*p* < 0.05).

Additionally, we examined the antiviral effect of neomycin administered in a therapeutic regimen: BALB/c and CBA mice were treated with 2.0 mg/25 µL of neomycin twice at 4 h and 24 h after being infected with influenza B virus. In this experiment, neomycin demonstrated no protective effect in either mouse line with respect to body weight dynamics or mortality compared with the PBS group (Figure S3).

### 3.4. A Single Prophylactic Intranasal Administration of Neomycin Provides Antiviral Protection Against the Influenza B Virus Sublethal Challenge in CBA Mice

The previous section demonstrated that prophylactic neomycin administration in CBA mice leads to reduced mortality compared to the control group in a lethal influenza B virus infection model. However, this effect was observed only as a trend and did not reach statistical significance. In the lethal infection model, animals were challenged with a high viral dose of 2 MLD50 (6.2 log_10_ EID_50_), at which the immunomodulatory effect of neomycin may be overwhelmed. Therefore, we investigated whether prophylactic neomycin administration affects pathogenic virus replication in the respiratory tract when mice are infected with a sublethal dose. The selected challenge dose was one that yields replication levels of the pathogenic strain in both the upper and lower respiratory tracts of 4.0–5.0 log_10_ TCID50/0.1 mL at the peak of virus shedding (days 2–4 post-infection) while remaining non-lethal.

**Figure 5:**
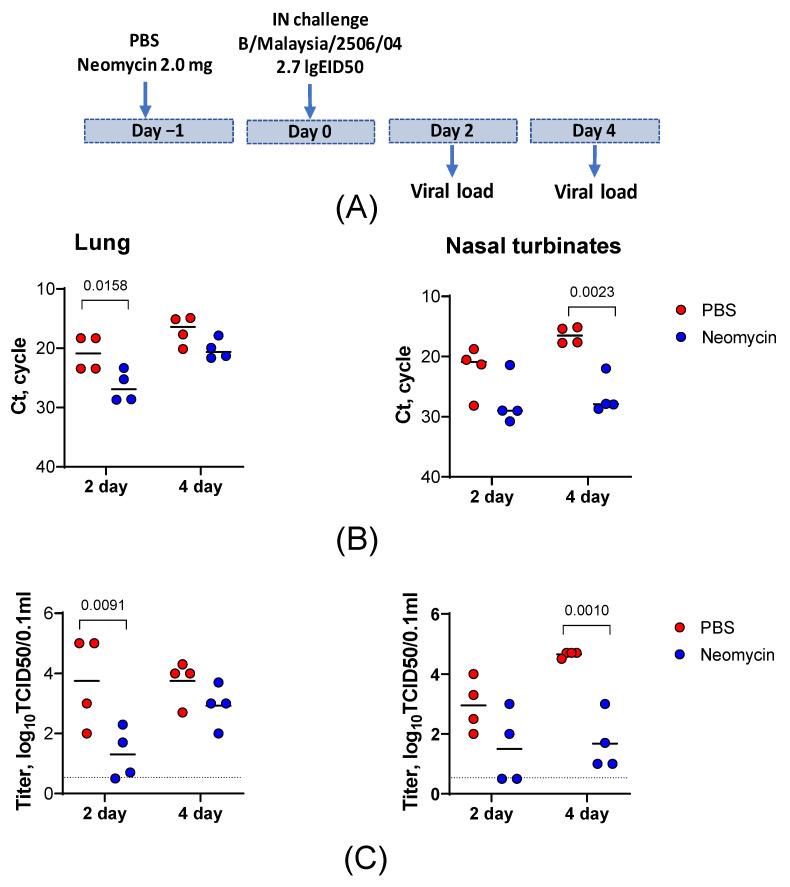
Prophylactic neomycin administration reduces the viral load in the respiratory tract tissues of CBA mice infected with a sublethal dose of influenza B virus. (**A**) Experimental design. CBA mice received a single intranasal dose of neomycin (2.0 mg; n = 8) or PBS (n = 8) in a volume of 25 µL, followed by intranasal infection with influenza B/Malaysia/2506/04 (10^2.7^ EID_50_) 24 h later. Viral RNA levels (**B**) and infectious virus titers (**C**) were measured in lung tissue and nasal turbinates on days 2 and 4 post-infection.

In this sublethal infection model, CBA mice received 25 µL of the neomycin solution (2.0 mg) or PBS 24 h before the challenge with influenza B/Malaysia/2506/04 virus at a 15 µL dose of 2.7 log_10_ EID_50_. Infectious virus titers and viral RNA levels were determined in lung and nasal turbinate homogenates on days 2 and 4 post-infection (Figure 5A).

A single prophylactic dose of neomycin administered 24 h before the challenge reduced viral titers in the lungs and nasal turbinates of the infected animals. On both days 2 and 4 post-infection, the viral loads in the nasal turbinates and lungs of neomycin-treated mice were lower than those in PBS-treated controls. In the lungs, neomycin significantly reduced infectious virus titers on day 2 post-infection, with a 282-fold decrease in the group mean titer (log_10_ 2.45, *p* < 0.05). On day 4, infectious virus levels in the neomycin-treated group remained lower than those in the controls, although the difference did not reach statistical significance. In the nasal turbinates, a significant reduction in infectious virus replication was observed on day 4 post-infection, with a 944-fold decrease in titer (log_10_ 2.975, *p* < 0.05) compared to the PBS-treated control group (Figure 5C). These virus shedding results were confirmed by RT-PCR (Figure 5B). Taken together, these data indicate that neomycin significantly reduces pathogenic influenza virus replication in the upper and lower respiratory tracts and was associated with lower viral titers at later time points.

### 3.5. Intranasal Neomycin Treatment Affects the Innate Immune Cell Composition in the Lungs of CBA Mice

To evaluate how intranasal administration of neomycin affects the composition of innate immune cells in the lungs, mice were injected with 2.0 mg of neomycin in a volume of 25 µL, and lung cellular composition was assessed at 24 and 48 h post-administration. CBA mice receiving an equivalent volume of PBS served as controls.

**Figure 6:**
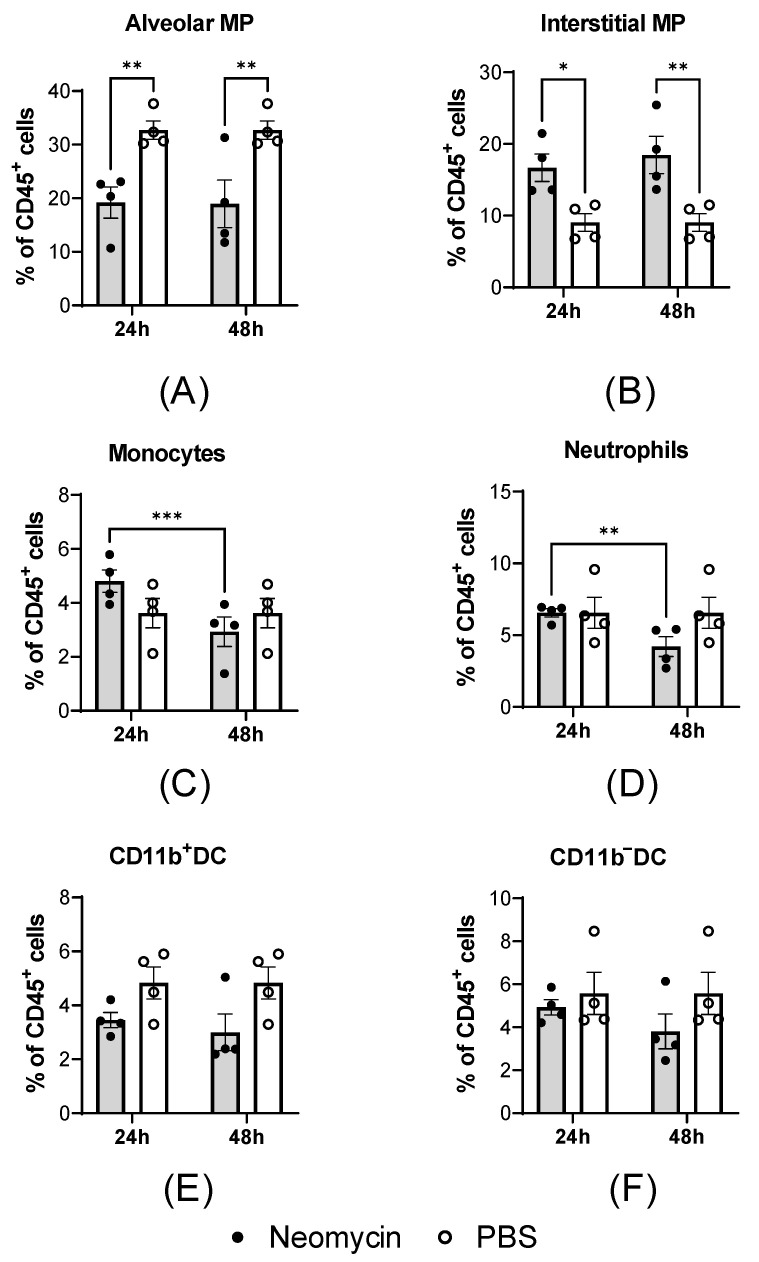
Effect of intranasal neomycin administration on the innate immune cell composition in the lungs of CBA mice. Neomycin (2.0 mg; n = 12) or PBS (n = 12) was administered intranasally in a volume of 25 μL. At 24 and 48 h post-inoculation, the innate immune cell composition was evaluated in single-cell suspensions prepared from lung tissue. Data are presented as the mean percentage of CD45^+^ cells ± SD, with dots indicating individual values for each animal: (**A**) alveolar macrophages, (**B**) interstitial macrophages, (**C**) monocytes, (**D**) neutrophils, (**E**) CD11b^+^ dendritic cells, and (**F**) CD11b^−^ dendritic cells. Statistical significance was defined as *p* < 0.05 by two-way ANOVA with Tukey’s multiple comparison test (*: *p* < 0.05, **: *p* < 0.01, ***: *p* < 0.001).

Intranasal neomycin administration induced a pulmonary cellular response in CBA mice, characterized by a decrease in the relative proportion of alveolar macrophages (MPs; Figure 6A) and an increase in interstitial macrophages (Figure 6B). This shift in cellular composition was observed at both 24 and 48 h post-administration. At the earlier time point, a trend toward an increased proportion of monocytes was also noted (Figure 6C); however, by 48 h, monocyte levels had returned to values comparable to those in control animals. Neomycin administration did not increase neutrophil proportions relative to controls at 24 h (Figure 6D); by 48 h, neutrophil counts had further declined compared to the 24 h time point. No significant differences in dendritic cell populations were detected between the neomycin- and PBS-treated groups (Figure 6E).

The observed cellular changes may be attributable to neomycin’s ability to mimic viral RNA, thereby activating endosomal and cytoplasmic pattern recognition receptors (TLR7/RIG-I-like). This activation triggers an IRF7-dependent antiviral gene expression signature, including OAS1 upregulation. This molecular signal may drive monocyte recruitment from the circulation into the lungs, where they subsequently differentiate into interstitial macrophages, thus accounting for the observed cellular shift. The absence of neutrophilia supports the interpretation that this response reflects an immunomodulatory effect rather than an infectious reaction.

## 4. Discussion

Influenza A and B viruses cause mild to moderate respiratory illness in most individuals, and severe cases account for no more than 0.4% of the total infections during seasonal influenza epidemics. For comparison, during the 1918–1919 influenza pandemic, the rate of severe disease was significantly higher, reaching 1.0% to 10% [14].

It is now established that the severity of influenza infection and its outcomes depend not only on viral properties but also on host-specific factors. Therefore, alongside etiotropic antiviral therapy, it is advisable to develop therapeutic strategies capable of activating the host’s innate defense mechanisms. Such approaches target signaling pathways that suppress viral replication or mitigate the inflammatory response, minimizing tissue damage and preserving organ function [15]. Animal studies have demonstrated that immunomodulatory drugs can reduce mortality without exerting direct antiviral effects or reducing viral replication. Research suggests that modulating inflammation in the body alone may be sufficient to improve disease outcomes. Immunomodulators can also provide a synergistic effect, enhancing the activity of antiviral drugs.

Neomycin, a broad-spectrum aminoglycoside antibiotic, is an approved topical medication indicated for the treatment of infectious inflammatory diseases of the mucous membranes, regardless of etiology. Although antibiotics are generally considered to be ineffective in treating and preventing viral infections, recent research has demonstrated that some antibiotics can contribute to the establishment of the antiviral state by modulating the host’s immune response [16]. In particular, neomycin has been previously shown to have an antiviral effect against respiratory viruses like influenza A/PR/8/34(H1N1), herpes simplex virus type 2, Zika virus, and SARS-CoV-2 [8]. Intranasal administration of neomycin in model animals induces strong interferon-stimulated gene (ISG) expression via the interferon-independent pathway, which leads to limited replication of influenza A and SARS-CoV-2 viruses and reduced mortality. Additionally, some ISGs can directly inhibit viral replication by degrading viral RNA, disrupting its transport, and blocking viral translation [17,18]. These findings highlight the potential of neomycin as a therapeutic or prophylactic agent against respiratory viruses.

In this study, we demonstrated that neomycin can activate the production of pro-inflammatory cytokines in human PBMCs, initiating an immune response characteristic of the early stages of infection. Specifically, neomycin triggered a pro-inflammatory response by stimulating IL1β, TNF-α, and IL6 secretion. Our data also revealed increased expression of GM-CSF, IFN-γ, and the anti-inflammatory cytokine IL10. Together, these effector molecules promote the polarization of CD4+ memory T cells toward a Th1 phenotype [19].

Interestingly, neomycin and poly(I:C) activated remarkably similar gene expression patterns. Poly(I:C) is known to act through the activation of the endosomal sensor TLR3 [20]. Supporting this mechanism, a study by Gopinath et al. using TLR3-deficient mice has shown that neomycin-induced ISG upregulation can also occur via the TLR3–TRIF–IRF3/7 signaling pathway [11].

Furthermore, in 2025, Salasaa et al. reported that neomycin exhibits high-affinity binding to phosphoinositides (PI4P) concentrated in the inner leaflet of the plasma membrane of eukaryotic cells [21]. We hypothesize that the interaction between neomycin and PI4P, a key regulator of cellular homeostasis, may define the ability of this antibiotic to induce inflammation. It is well-established that the balance between inflammatory processes and the mechanisms that constrain them is critical to the pathogenesis of infectious diseases and directly influences disease outcomes [22].

Our findings suggest that neomycin activates ISGs via TLR3 signaling in a manner similar to poly(I:C), and its mechanism of action also involves the PI4P pathway.

In this study, we investigated how intranasally administered neomycin affects the antiviral state and the progression of influenza B virus infection in two laboratory mouse strains—BALB/c and CBA—that, like most inbred mice, carry a defective Mx1 gene [23]. This defective phenotype can be caused by a nonsense mutation, as in CBA/J mice, or a deletion leading to a reading frame shift with the formation of a stop codon, as in BALB/c mice [24]. Lacking a functional Mx1 protein, these mouse lines are highly susceptible to influenza A and B viruses. Our study demonstrated that in mice of both strains, intranasal administration of neomycin induced multiple ISGs not only in the respiratory tract but also in the spleen.

In BALB/c and CBA mice, neomycin significantly increased IRF7 expression in the lungs and the spleen. Previous research has established IRF7 as a critical player in antiviral immunity and an important regulator controlling influenza A virus spread [25]. IRF7 knock out in MDCK cells leads to enhanced replication of the vaccine strain, while reduced IRF7 expression in dendritic cells impairs viral pathogen recognition and antiviral responses [26]. Recent studies have further shown that innate defects in human IRF7 can predispose individuals to severe influenza [27,28].

Furthermore, we demonstrated that neomycin treatment increased the mRNA levels of the endosomal receptor TLR7 in the splenic tissue of both mouse strains. Endosomal sensors, such as TLR7, detect single-stranded RNA during viral infection and trigger downstream signaling via the TLR7/MyD88 pathway [29]. This, in turn, induces the production of pro-inflammatory cytokines and IFN-α by plasmacytoid dendritic cells, enabling innate immune cells to recognize and respond to infections caused by RNA viruses.

We further investigated whether neomycin influences the course and outcome of influenza B virus infection in mice. Therapeutic administration of neomycin (given against the background of established infection) aggravated disease severity in both mouse lines. It is worth noting that the additional fluid volume administered may have exerted a confounding mechanical effect, as mortality in the PBS-treated therapeutic control group was also higher than that observed with prophylactic administration. Nevertheless, neomycin administration under therapeutic conditions worsened the course of influenza B in both CBA and BALB/C mice. This is possibly based on the upregulation of the innate immune response by neomycin in addition to the influenza B virus infection.

Prophylactic intranasal administration of neomycin revealed strain-specific differences in lethal disease severity between BALB/c and CBA mice. Neomycin-treated CBA mice showed a moderate reduction in body weight loss and mortality that was not observed in BALB/c mice. After analyzing pro-inflammatory factors in response to neomycin, we observed increased OAS1 expression in the lungs and spleen of CBA mice; however, no OAS1 transcript was detected in BALB/c mice, either infected, neomycin-treated, or intact. OAS proteins are known to have antiviral properties against various viruses, including SARS-CoV-2, African swine fever virus, hepatitis B virus, and porcine reproductive and respiratory syndrome (PRRS) virus [30]. The increased disease severity observed in BALB/c mice may therefore reflect an increased inflammatory response (immunopathology) rather than the absence of a direct antiviral effect *per se*. OAS1 and RIGI are key sensors of viral RNA that act in concert to provide a multilayered antiviral response. Notably, OAS1 can also function as a negative regulator of immune activation by suppressing ISG expression [31]. The increased RIGI expression may be a consequence of the absence of OAS1-mediated regulation in BALB/c mice, and the resulting loss of mutual regulatory control may predispose these animals to immunopathology and contribute to the increased disease severity observed in this strain.

A single prophylactic dose of neomycin reduced weight loss and mortality in the lethal infection model, although neither effect reached statistical significance. We were also unable to detect any significant effect on viral load in the lower respiratory tract, which may be due to the supraphysiological challenge dose used to model lethal infection. In the sublethal influenza B infection model, however, prophylactic neomycin administration significantly reduced virus replication in both the upper and lower respiratory tracts. Lung virus titers decreased by approximately 2 log_10_ on day 2 post-infection, and nasal turbinate titers decreased by approximately 3 log_10_ on day 4 post-infection relative to PBS-treated controls. These findings indicate that the immunomodulatory effect of neomycin can limit viral replication, although this advantage was likely obscured in the lethal model by the excessively high challenge dose.

To elucidate the cellular mechanisms underlying the antiviral response, we investigated the effect of neomycin on the innate immune cell composition in the lungs of CBA mice. Intranasal neomycin administration induced a specific pulmonary immune response, characterized by a decrease in the proportion of alveolar macrophages and a concomitant increase in interstitial macrophages, which was accompanied by a transient influx of monocytes (at 24 h post-administration). No signs of acute inflammation were observed, as neutrophil levels did not increase and dendritic cell proportions remained unchanged [32]. This suggests that the antiviral effect of neomycin is mediated through immune modulation [33] rather than direct inhibition of viral replication.

Overall, the findings indicate that prophylactic intranasal neomycin administration establishes an antiviral state which has the potential to attenuate the severity of influenza B. These effects point to underlying immunomodulatory mechanisms that should be taken into account when developing novel therapeutic approaches.

## Data Availability

The original contributions presented in this study are included in the article/Supplementary Materials. Further inquiries can be directed to the corresponding authors.

## Supplementary Materials

The following supporting information can be downloaded at: https://www.mdpi.com/article/10.3390/v18040444/s1, Figure S1: Determination of the lethal dose in BALB/c mice (MLD_50_); Figure S2: Gating strategy for the assessment of major innate immune cell populations in the lungs; Figure S3: Production of IFNα2 (a), IFNβ (b) and IFNλ2 (c) in PBMCs after 24 h of stimulation with neomycin (2 mg/mL) or poly(I:C) (10 μg/mL); Figure S4: The effect of the therapeutic administration of neomycin in CBA and BALB/c mice on influenza B virus infection severity; Table S1: Primer and probe sequences used to assess mouse gene expression; Table S2: Primer and probe sequences used for assessing gene expression in human cells.
