# Supplementary material for: Neomycin Exhibits Immunomodulatory and Antiviral Activity Against Influenza B Virus
Source: Viruses. 2026 Apr 7;18(4):444. doi: 10.3390/v18040444 (PMC13120434; doi:10.3390/v18040444)

Supplementary 1

Figure S1. Determination of the Lethal Dose in BALB/c Mice (MLD_{50}). Six- to eight-week-old BALB/c mice (n=20) were intranasally under light ether anesthesia inoculated with 30 μl of virus B/Malaysia/2506/04 at the dilutions (5 animals for each dilution of virus stock) indicated in figure legends. Subsequent to infection, survival was monitored for up to 14 days. Mouse LD_{50} value was calculated according to the method of Reed and Muench (1938).

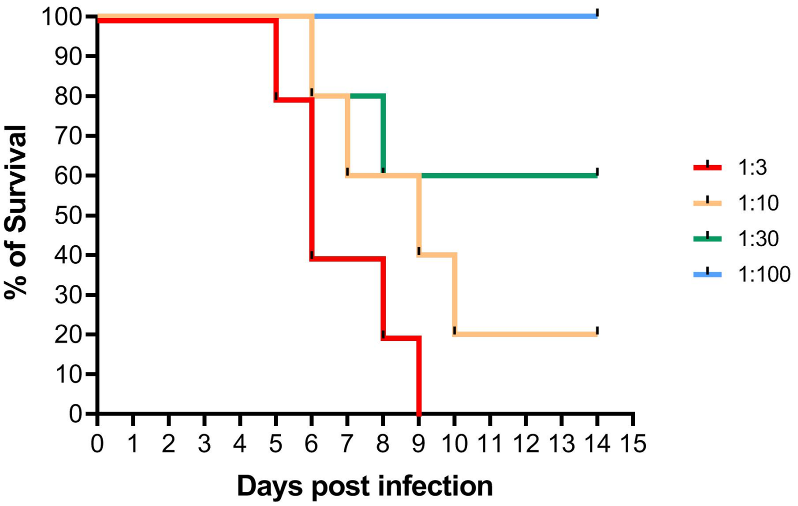

Supplementary 2

Table S1. Primer and probe sequences used to assess of mouse gene expression.

Gene	Primers/Probe	Sequence
**OAS1ag**	Forward	GATGTGCCGACGGTGGT
	Reverse	TGGACAGGAGTCAAACATGGC
	Probe	(**Quasar705**)-CACCTGGCATCAGACTCCGTGCTTCT-(**BHQ2**)
**IRF7**	Forward	CCTGGAAGCATTTCGGTCGGT
	Reverse	CTCTTCGCTCTCTTCGCTCA
	Probe	(**HEX**)-CCACCTAGTGGAGTTAACCTGCCA-(**BHQ1**)
**TLR7**	Forward	TCATGTGCCATGCTCAGTT
	Reverse	GGCAGATGTGTGGCTCTTAT
	Probe	(**FAM**)-TGTGCCTAGGAGACAACACAAGGC-(**BHQ1**)
**RIG I**	Forward	CTGTATAGCTTTGGCTGTCCT
	Reverse	CGCCTTTAATCCCAACACTTG
	Probe	(**FAM**)-ACTCAGAAATCCGCCTTCCTCTGC-(**BHQ1**)
**Mda5**	Forward	GGTTCAGGCTTGCTTCTTCT
	Reverse	ACTCCCTTCATCATAAGAGATGATTAG
	Probe	(**FAM**)-TCTTCTGCAAACACAGTACCATCCTGG-(**BHQ1**)
**TLR3**	Forward	TGGTCACCAACTGGCTATTAAA
	Reverse	CCATCTATCACTGTGGCTCTTC
	Probe	(**FAM**)-ACCCATGCCTGAGTAGTCTTCTCTGA-(**BHQ1**)
**TLR8**	Forward	AGGCAGCTTATTATGCCTACTT
	Reverse	TGTATCAACTTCACCAGCATCT
	Probe	(FAM)-TCAATCCCTAAGAACATTTGCCACTGT-(**BHQ1**)
**Mx2**	Forward	GTTAAGCTGGCTCTGTCCTT
	Reverse	AGCATAAAGGCTGGTGGTATAG
	Probe	**(HEX**)-TTGTGATTCAGGGACAGAAGGGCT-(**BHQ1**)
**IL6**	Forward	TGATGGATGCTACCAAACTGGA
	Reverse	CTGAAGGACTCTGGCTTTGTCT
	Probe	**(ROX)**-CTTCTGGAGTACCATAGCTACCTGGAGTA-(**BHQ2**)
**HPRT1**	Forward	GAAGCTCTCGATTTCCTATCAGT
	Reverse	CAACGATTTACTGAAAGTGGGAAA
	Probe	**(HEX**)-ACATGTTTCAGCAGTGTTGGCTGT-(**BHQ1**)
**Rplp0**	Forward	CAAAGGAAGAGTCGGAGGAATC
	Reverse	CTTTCTCAAATTAAGCAGGCTGAC
	Probe	**(ROX)**-TCTTCGACTAATCCCGCCAAAGCA-(**BHQ2**)
**Ubc**	Forward	CCCAGTGTTACCACCAAGAAG
	Reverse	CCCATCACACCCAAGAACAA
	Probe	**(Cy5)**-AGACAGACGTACCTTCCTCACCACA-(**BHQ2**)
**GAPDH**	Forward	AATGGTGAAGGTCGGTGTG
	Reverse	ACAAGCTTCCCATTCTCGG
	Probe	**(HEX)-**TTGACTGTGCCGTTGAATTTGCCG**-(BHQ1)**

Supplementary 3

Table S2. Primer and probe sequences used for assessing gene expression in human cells.

Gene	Primers/Probe	Sequence
MxA	Forward	GAGACAATCGTGAAACAGCAAATCA
	Reverse	TATCGAAACATCTGTGAAAGCAAGC
	Probe	(**HEX**)-CACTGGAAGAGCCGGCTGTGGATATG-(**BHQ1**)
OAS-1	Forward	CCAAGGTGGTAAAGGGTGGCT
	Reverse	CTGGACCTCAAACTTCACGGAAA
	Probe	(**Cy3**)-AGGCCGATCTGACGCTGACCTGGTTGT-(**BHQ2**)
IL1b	Forward	AGCTGATGGCCCTAAACAGA
	Reverse	TGGTGGTCGGAGATTCGTAG
	Probe	(**HEX**)-GCCCTCTGGATGGCGGCATC-(**BHQ1**)
IL10	Forward	TCCCTGTGAAAACAAGAGCAAG
	Reverse	CTCATGGCTTTGTAGATGCCT
	Probe	(**HEX**)-CCGTGGAGCAGGTGAAGAATGCC-(**BHQ1**)
IL6	Forward	CCACTCACCTCTTCAGAACG
	Reverse	CATCTTTGGAAGGTTCAGGTTG
	Probe	(**HEX**)-AAATTCGGTACATCCTCGACGGCATC-(**BHQ1**)
IL18	Forward	AAACTATTTGTCGCAGGAATAAAGAT
	Reverse	GCTTGCCAAAGTAATCTGATTCC
	Probe	(**ROX**)-TGCAATTGTCTTCTACTGGTTCAGCAGC-(**BHQ2**)
NfkB	Forward	GCTCAGTGAGCCCATGGAAT
	Reverse	TGATGCTCTTGAAGGTCTCATATGTC
	Probe	(**FAM**)-TCACCGGATTGAGGAGAAAC-(**BHQ1**)
Rig-I	Forward	GAGCACTTGTGGACGCTTTA
	Reverse	ATACACTTCTGTGCCGGGAG
	Probe	(**ROX**)-CCTGGCATATTGACTGGACGTGGC-(**BHQ2**)
MDA5	Forward	AAACCCATGACACAGGAATGAACA
	Reverse	TGTGAGCAACCAGGACGTAG
	Probe	(**Quasar705**)-CACAGTGGCAGAAGAAGGTCTGGA-(**BHQ2**)
GAPDH	Forward	CAGTCAGCCGCATCTTCTTTTGCGTCG
	Reverse	CAGAGTTAAAAGCAGCCCTGGTGACCAGG
	Probe	(**FAM**)-TGGGGAAGGTGAAGGTCGGAGTCAACGGATTTGGTC-(**BHQ1**)

Supplementary 4

Figure S2. **Gating strategy for the assessment of major innate immune cell populations in the lungs.** After excluding doublets using FSC-A/FSC-H (not shown) and gating on live single cells based on light scatter characteristics (FSC-A/SSC-A -Cells) and Zombie Red dye exclusion (Live cells), immune cells were gated for the CD45 marker (CD45+). Subsequent analysis identified the following cell populations: neutrophils – CD45^{+}MHCII^{-}CD11b+Ly6G^{+}; monocytes – MHCII^{-}CD64^{+}CD24^{+}; alveolar macrophages – MHCII^{+}CD64^{+}CD11c^{+}CD11b^{-}; interstitial macrophages – MHCII^{+}CD64^{+}CD11b^{+}CD11c$^{+/-}$; dendritic cells type 1 (CD11b^{-}DC) – CD45^{+}CD11c^{+}CD11b^{-}MHCII^{+}CD64^{-}CD24^{+}; and dendritic cells type 2 (CD11b^{+}DC) – CD45^{+}CD11c^{+}CD11b^{+}MHCII^{+}CD64^{-}CD24^{+}. Other abbreviations: nonAPC – non-APC; APC – antigen-presenting cells; DC – dendritic cells; MP – macrophages.

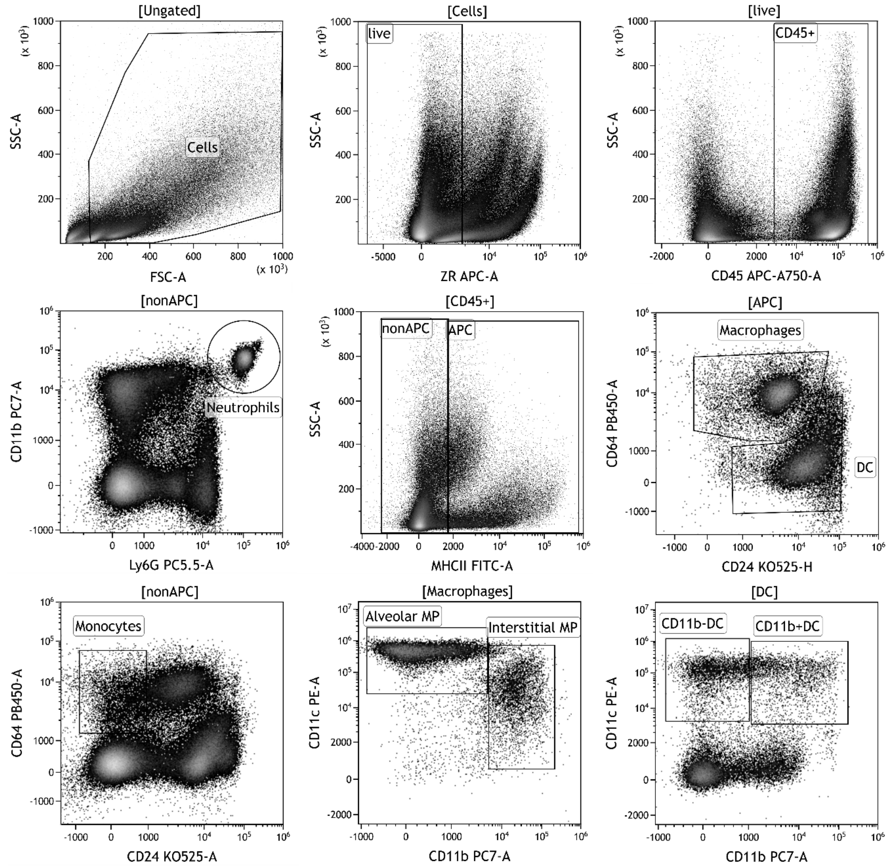

Supplementary 5

Figure S3. **Production of IFNα2 (a), IFNβ (b) and IFNλ2 (c) in PBMCs after 24 hours after stimulation with neomycin (2 mg/mL) or poly(I:C) (10 μg/mL).** Protein production was measured in a PBMC cellular medium after 24 hours of neomycin (2 mg/ml) or poly(I:C) (10 mg/ml) stimulation. Intact cells were used as a control (CC). The horizontal lines show the average cytokine concentrations in the cellular supernatant (pg/ml). The individual symbols represent the individual values for each repetition. The statistical significance was assessed by the nonparametric Kruskal-Wallis test with the Dunn correction for multiple comparisons (ns – $p>0.05$;* – $p < 0.05$; ** – $p < 0.01$; *** – $p < 0.001$, **** – $p < 0.0001$).

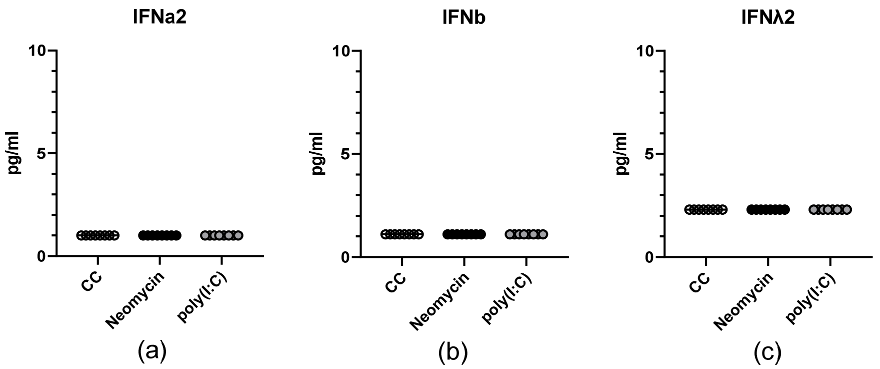

Supplementary 6

Figure S4. **The effect of the therapeutic administration of neomycin in CBA and BALB/c mice on the influenza B virus lethal infection severity.** (A) Design of the experiment. CBA and BALB/c mice were intranasally infected with influenza virus B/Malaysia/2506/04 (2 MLD_{50}). Than twice on 4 and 24 hours following challenge animals were given a intranasal dose of 2 mg of neomycin or PBS in a volume of 25 μL. The animals' body weight (B) and survival (C) were monitored daily for 14 days after infection. If an animal lost more than 30% of its initial body weight, it was sacrificed in accordance with ethical guidelines. On the 2rd day after challenge the infectious virus titers (D) were measured in lung tissue.

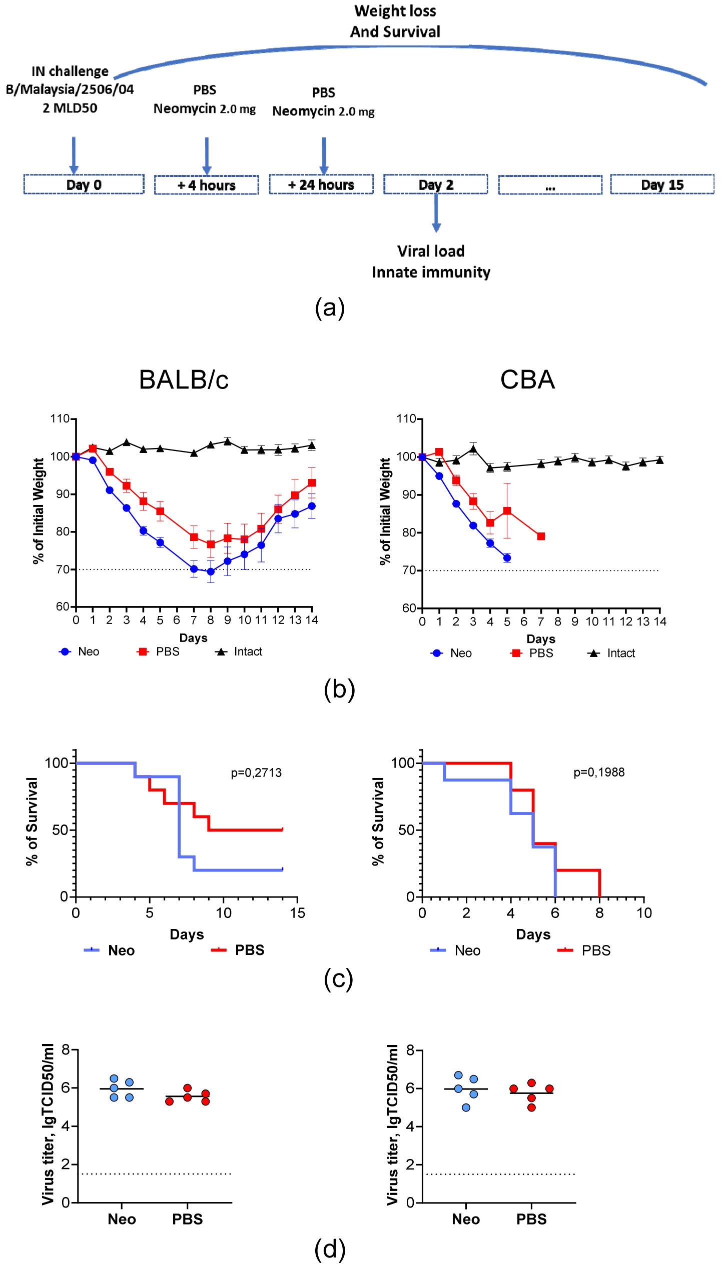

Supplement: Supplementary file 1 [file viruses-18-00444-s001.zip › viruses-4226113-supplementary.pdf]
